# Initial Impact of Different Feeding Methods on Feed Intake Time in Stabled Icelandic Horses

**DOI:** 10.3390/ani14081211

**Published:** 2024-04-18

**Authors:** Sveinn Ragnarsson, Sigríður Vaka Víkingsdóttir, Guðrún Jóhanna Stefánsdóttir

**Affiliations:** Department of Equine Science, Hólar University, 551 Sauðárkrókur, Iceland; svaka@holar.is (S.V.V.); gudrunst@holar.is (G.J.S.)

**Keywords:** feed intake time, Icelandic horses, feeding methods, haylage, hayball, haynet, box floor, metal corner manger

## Abstract

**Simple Summary:**

The natural behaviour of horses is to spend the majority of their time foraging. The feed intake time of stabled horses is often far from that, since their feed intake is limited to their nutritional requirements in order to avoid overfeeding and obesity. To approach their natural foraging time, it is important to find methods which can extend the feed intake time for stabled horses. The aim of this study was to estimate if different feeding methods could extend horses’ feed intake times. We measured how long it took for four Icelandic horses to eat 7 kg of high-energy haylage (3.5 kg/meal), from a haynet, hayball, manger, and straight from the box floor, for one day per method. To record the horses’ feed intake time, a video surveillance system was applied using two cameras. All horses stayed healthy throughout the study and ate all feed that was offered. From this study, it can be concluded that feeding high-energy haylage in a hayball or in a haynet can increase the feed intake time of maintenance-fed horses by 13% per day, as compared to more traditional methods. Thus, with simple feeding methods, it is possible to extend the feed intake time of stabled horses, thereby closer resembling horses’ natural foraging time.

**Abstract:**

The natural behaviour of horses is to spend the majority of their time on feed intake The feeding of stabled horses is, however, often far from that, as their feed intake is limited to their nutritional requirements. In order to approach their natural foraging time, it is important to extend the feed intake time of stabled horses. The aim of this study was to estimate if the feed intake time differs when feeding haylage in a haynet, hayball, metal corner manger, or from the box floor. The experimental design consisted of a Latin square, occurred across four days with four adult Icelandic horses and four treatments. Horses were stabled in individual boxes and fed 7 kg of high-energy haylage in two even meals while the intake time was recorded. The feed intake time per kg DM was shorter from the manger or the box floor than from a haynet or hayball (81 or 85 min versus 94 or 96 min; *p* < 0.05). It can be concluded that feeding haylage in a hayball or in a haynet can increase the feed intake time by 13% per day (12 min/kg DM/day) when compared to the more traditional methods. Thus, with simple alternatives, it is possible to extend the feed intake time of stabled horses.

## 1. Introduction

The horse has evolved for millions of years as a herbivore, specifically through its behaviour and digestive physiology [1]. Modern horses mostly choose grass for eating [1], ingesting small amounts of grass continuously [2]. The natural behaviour of horses is to roam freely and to spend the majority of their time grazing and/or eating, if allowed free access to roughage [3,4].

The feeding management of stabled horses is often far from enough to fulfil the natural eating behaviour of horses, as the feed intake time is limited by the nutritional requirements, the amount of roughage being fed, and the number of meals [5]. Horses are often fed a combination of high-energy grain meals and little forages, which are sometimes offered only two times per day, thus leaving the horses for relatively long periods without feed [6,7]. Limiting the duration of the feed intake time of horses and feeding a diet which is low in fibre and high in grains can increase the risk of undesirable and/or stereotypic behaviour, often seen as an indicator of poor welfare, e.g., chewing wood and crib-biting [5,8] or ingesting wood shavings and/or performing coprophagy [2,5,9]. Stereotypic behaviours can also be developed due to a lack of social contact and physical exercise in stabled horses. Examples of frequently observed locomotion stereotypes are weaving and box walking [10]. The horse is a grazing social herbivore, adapted to eat plant fibre- and forage-based diets [11]. In a study by Halldórsson [12], it was common to feed 6.4 kg (approx. 70% dry matter content) of rather late-cut, plastic-wrapped forage to stabled horses in Iceland. The maturity stage and cutting time of grass plants can have a great effect on the energy level and the amount of fibre in the forage, as early-cut grass (June) has a higher energy content and a lower fibre content than late-cut grass (July, August) [13,14]. Feeding horses late-cut forage and more mature grass means that it is possible to feed a higher amount in order to fulfil the energy requirements, thus lengthening the feed intake time [13]. However, the country of origin may influence the nutritive value of particular forages [15], and Icelandic haylages seem to be rather high in energy, despite being late cut. In recent studies [14,16], the energy values (digestible energy) were relatively high when compared to reported values [17], meaning less amounts are needed to fulfil horses’ energy requirements [18]. The nutrient requirements of Icelandic horses for energy and protein are similar to the international minimum criteria for horses [17,18], referred to as “easy keepers” [19]. In Iceland, feed units (Fu) have been used for decades and are still used to evaluate the energy content of feeds and the requirements of horses. Icelandic forages are considered to provide very high energy (high quality) to horses when containing 0.70–0.80, high energy when containing 0.65–0.72, medium when containing 0.60–0.66, and low energy when containing 0.50–0.59 Fu in kg DM [20]. The criteria for energy content overlaps as the protein content is also considered in the classification, adapted from [20]. The average energy content of forages analysed for horses in Iceland over the last 4 years (2020–2023) was 0.73 Fu (ranging from 0.55–0.87, unpublished data, Efnagreining.is, 2024; personal communication). Overfeeding seems to be increasing the problems for riding horses and is of grave concern, as recent studies [21,22] have shown that obesity is becoming a major welfare issue [23], not least in ridden leisure horses and ponies. Therefore, it is important to find ways to extend the feed intake time of stabled Icelandic riding horses when fed high-energy forages in smaller amounts, often resulting in a lower dry matter intake than recommended [9]. In addition to affecting the length of the feed intake time by controlling the quality (energy-level) of the forage, it is also important to find feeding methods which can extend the feed intake time [24]. Several studies [24,25,26,27,28,29] have used different “slow feeding” bags/devices/equipment or haynets to decelerate the eating rate of horses by making it more time consuming for them to access the forage. The idea behind using such slow-feeding methods is to lengthen the feed intake time of the horses, and thus to get closer to recreating their natural foraging time. Feeding hay from a haynet is one of the feeding methods which has been used to increase the feed intake time of horses [24,26]. Haynets can be made of different fabrics in different sizes with different sized holes/openings, through which horses draw out and eat the forage [27]. Earlier studies [24,26] have shown that the feed intake time of horses is longer when eating forage from a haynet when compared to eating it from the stable floor. Rochais et al. showed that the use of either a haybag or a special “slow-feeder”, described as a plastic container with holes on which a plate descended as the hay was consumed, could lengthen the feed intake time of horses, as compared to eating the forage from the floor [27]. Correa et al. found that a specially designed “slow-feeding hay bag” with a capacity of 5 kg of hay and 45 mm squared openings on two sides could lengthen the feed intake time of horses [28]. According to the authors´ knowledge, measurements of the feed intake time of haylage in stabled Icelandic horses which compare different feeding methods have not yet been published. The aim of this study was to measure feed intake time of maintenance-fed Icelandic horses consuming same amount of high-energy haylage via the use of different feeding methods, from a metal corner manger, a box floor, a haynet, or a hayball.

## 2. Materials and Methods

The research was performed at Hólar University 15–18 of December 2022.

### 2.1. Horses

Four adult Icelandic horses (two mares and two geldings) were used for the study. Their age was 12 ± 3 years, their height was at withers 142 ± 4 cm (measuring stick), body weight (BW) was 406 ± 5 kg (Smartscale 300, Gallagher, Hamilton, New Zealand), and body condition score (BCS) was 3.5 [30], corresponding to approximately BCS 6–7 on the Henneke scale [31]. Before the study, all horses had been fed similar amounts (6–7 kg/day) of haylage for 2–3 months and had been stable-fed 3 times a day in a metal manger. During that period, the horses were housed in individual boxes, with the possibility to touch other horses and to go out in a group with other horses daily. The horses were used for riding lessons at the University (except during the experiment) and had been trained 4–5 times a week for the last 3–4 months. They had not been grazing for 2–3 months before attending the study.

### 2.2. Preparation of the Experiment and Forage Compositions

The experiment was performed in the experimental stable at Hólar University. The 4 horses were moved to the stable 3 days prior to the start of the study in order to adapt to the experimental condition. For these 3 days, the horses were trained to eat from a haynet and a hayball, ensuring they would eat the haylage and become adapted to the feeding methods. The horses had been fed with the haylage (Table 1) for several weeks before starting the experiment. 

One type of haylage with a mixed botanical composition was fed to the experimental horses, harvested on the 19th of July 2022 at the Hólar University farm in north Iceland. The haylage was analysed in a commercial laboratory [32] using near-infrared reflectance spectroscopy (NIRS) prior to starting the experiment. The dry matter (DM) content and nutritive value is given in Table 1.

### 2.3. Experimental Design and Feeding Methods

**Figure 1:**
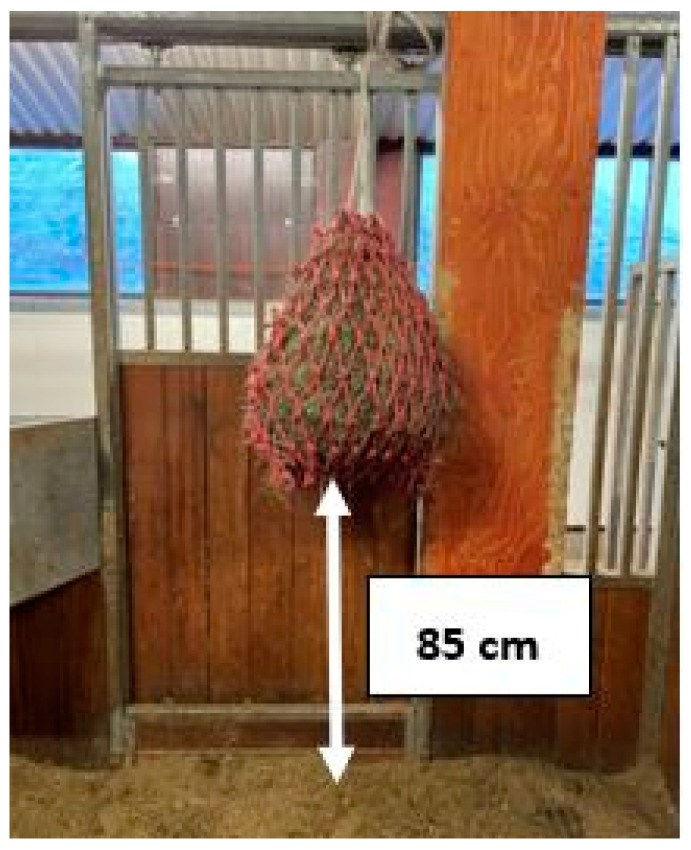
Haynet filled with 3.5 kg of haylage.

**Figure 2:**
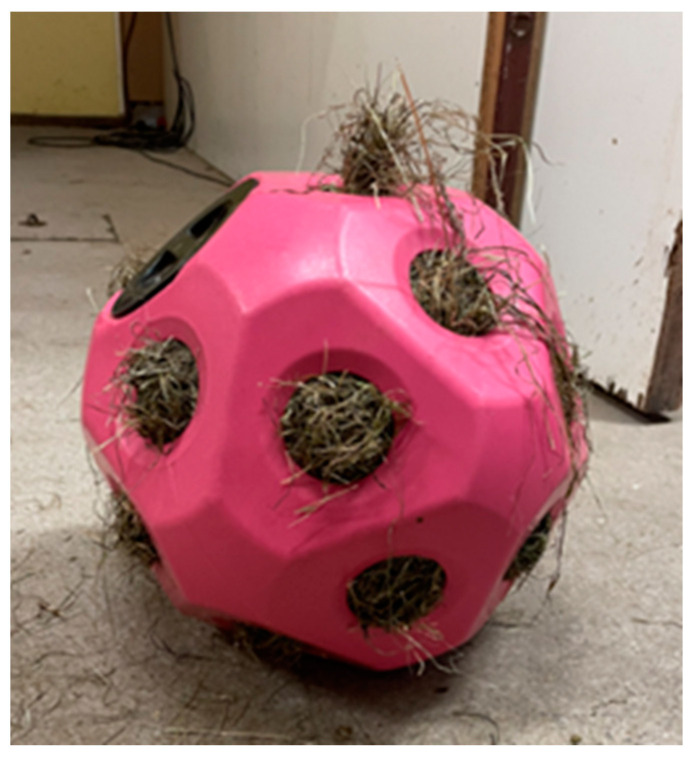
Hayball filled with 3.5 kg of haylage.

**Figure 3:**
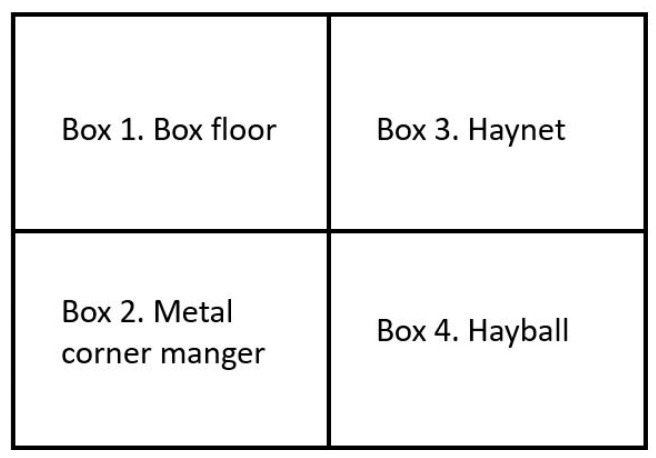
Arrangement of feeding methods in the stable boxes.

The study lasted 4 days, and the experimental design consisted of a Latin Square where 4 horses were randomly divided into 4 treatments as follows: (1) from the box floor, (2) from a haynet (hole sizes 10 × 10 cm), located 85 cm from the box floor (Figure 1), (3) from a hayball (19 holes of 7 cm in diameter, 38 cm high and empty weight 1.48 kg) (Figure 2); and (4) from a metal corner manger (depth 40 cm and height from floor 104 cm). During the experiment, the horses were fed two 3.5 kg meals of haylage at 07:30 and 18:15. It took on average 40–50 s to weigh every 3.5 kg per meal in a plastic IKEA bag, and an additional 20–30 s to fill the haynet, or 2½–3 min to fill the hayball. Video surveillance using 2 cameras located in the roof was used to record the horses’ feed intake time, and the data were stored on a computer (SmartPSS, Dahua Wiki, Mexico City, Mexico). The feed intake time was measured from the video recordings. During the experimental and preparation period, horses were outside daily between 12:30 and 13:30, and were fed 100 g of a mineral and vitamin supplement (Reformin Plus, Höveler, Münster, Germany) at 13:30. The horses were individually housed in 2.37 m × 3 m (width × length) boxes, and each horse could see the other 3 horses (Figure 3). In the boxes, there was a permanent bed, approximately 10 cm thick, based on wood shavings from pellets.

During the experiment, each horse was fed using each feeding method for one day, i.e., two meals (morning and evening). The horses were moved between the boxes so that each method was always applied in the same box. The horses were moved to their boxes 30 min before receiving the morning meal. The feed intake time was registered from the start of the eating period and was assumed to be finished when the horses had not been eating for 5 continuous minutes. No measurable leftovers were detected, and only a few straws remained after each meal.

### 2.4. Heart Rate Measurements

During the experimental period, the heart rate of each horse was recorded during the feed intake time. The heart rate monitors (Polar Vantage M with H10 elastic band, Polar Electro, Kempele, Finland) were placed on the horse approximately 45 min before the start of each meal, and then removed approximately 45 min after finishing their meal. The heart rate monitors were set to record in a 1 s mode. The horses were all accustomed to heart rate monitoring before the start of the experiment. The resting heart rate of the horses was estimated during a 10 min period (between 15th–25th min) following the beginning of the recording before each meal. The heart rate recordings were successful, except only for two measurements during rest due to a loss of contact, but it was noticed before starting heart rate recording during feeding on these two occasions.

### 2.5. Statistical Analysis

All data regarding the feed intake time were registered in Excel (2019, Microsoft Headquarters, One Microsoft Way, Redmond, WA, USA.), and then transported for analysis in SAS 9.4 (SAS Institute, Cary, NC, USA). The Proc Mixed model Y_ijk_ = µ + α_i_ +β_j_ + a_k_ + e_ijk_, was applied. Y_ijk_ is the response (feed intake time in seconds) or heart rate in bpm (average, peak, during 30 min after finishing eating); µ is the mean value; α_i_ is the fixed effect of the treatment; β_j_ is the fixed effect of the day; a_k_ is the random effect of the horse; and e_ijk_ is the residuals. The results were expressed as least squares means (LSMeans) with a standard error (±SE), unless otherwise stated. Paired Student´s *t* test was used to compare the resting heart rate of the horses to the average heart rate during the feed intake time and during the 30 min after eating. The level of significance was set to *p* < 0.05.

## 3. Results

### 3.1. Feed Intake Time

The execution of the study was successful, the experimental horses were healthy with a good appetite, and all four horses finished all treatments. The horses were fed close to the maintenance level for easy keepers [14,16,17] on energy bases or 7 kg/day, which corresponds to approximately 1% of DM per BW or 2.82 Fu, and approximately 360 g of digestible crude protein (DCP), corresponding to 25% more than the requirements (3g DCP per kg BW^0.75^) [33]. The feed intake time was longer when using the hayball and the haynet when compared to the metal corner manger and the box floor (Table 2). There was no difference (*p* > 0.05) in the feed intake time between the horses or days.

### 3.2. Heart Rate

The resting heart rate of the horses was on average 31 ± 2 bpm (mean ± SD, range: 26–36 bpm, number of measurements = 30). The average heart rate of the horses during the feed intake time was higher when eating from the box floor than from the metal corner manger and from the haynet (44 ± 1 vs. 43 ± 1, 42 ± 1 bpm, *p* < 0.05), but was not different from the hayball (44 ± 1 vs. 44 ± 1 bpm; *p* > 0.05). There was no difference between the feeding methods in terms of the peak heart rate during eating (metal manger, box floor, haynet, and hayball: 68 ± 8, 77 ± 8, 65 ± 8, and 69 ± 8 bpm; *p* > 0.05), nor during the 30 min after eating (all feeding methods: 37 ± 1 bpm; *p* > 0.05). On average, the heart rate of the horses was 12 ± 3 bpm (mean ± SD) higher (*p* < 0.001) during the feed intake time when compared to the resting heart rate. The heart rate during the 30 min after eating was higher than the resting heart rate (mean ± SD: 37 ± 2 vs. 31 ± 2; *p* < 0.001).

## 4. Discussion

The results showed that it is possible to extend the feed intake time of stabled Icelandic horses by 13% per day (approximately 12 min/kg DM) via the use of a haynet or hayball, as compared to feeding via the use of a metal corner manger or the box floor. These results are in accordance with results from earlier studies that used a haynet [25,26,29], or that used a specially designed bag with holes [28] and a container with a plate with holes which descends as the hay is consumed [27,34]. Our results suggest that, by applying the easy-to-use feeding methods (hayball and haynet), it is possible to closer approach the horses’ natural foraging time when feeding high-energy haylage at a maintenance level. However, the horses in our study only spent on average 5–6 h/day (21–25% of 24 h) on feed intake. For comparison, it should be considered that the natural behaviour of horses is to spend 8.5–12 h per day on foraging-related behaviours. When stabled and if only allowed limited access to foraging, they could fill up their time by eating bedding and/or practicing coprophagy [2,9], and might also practice stereotypic behaviours like wood chewing and crib-biting [35,36]. The horses in our study stayed calm, and the caretakers did not observe any visible signs of stress, stereotypic behaviours, or frustration. Interestingly, the average heart rate of the horses during the feed intake time was not higher when eating from the hayball and haynet when compared to when eating from the box floor or the metal manger. There was indeed very little difference observed in the average heart rate between the feeding methods (1–2 bpm higher from box floor than from haynet or manger), and the biological relevance of that difference is questionable. However, on average, the heart rate of the horses increased during the feed intake time and for 30 min afterwards, indicating increased physical activity and digestion. The average heart rate of the horses in our study during the 30 min after eating was very similar to values found in Brazilian jumping horses (36 ± 4 bpm) when fed from slow feeder hay bags [28].

In our study, the feed intake time per kg of haylage fed was 44–52 min. Some earlier studies [25,26,29,34] have measured the feed intake time of forages in horses using different feeding methods, being in the range 25–78 min/kg forage. It is important to consider that many factors can affect the feed intake time, e.g., DM% of the forage, botanical composition of the forage, feeding method (e.g., size of holes in the haynet, hayracks, and how multiple haynets are used); also, the size of the horses can affect the biting size and, thereby, the feed intake rate. Ellis et al. [25] measured horses’ feed intake time from a haynet when fed forage with two different DM content (89% and 68%), ranging from 25 to 33 min/kg hay, and they found that after a 9–10 day period of eating from haynets, the horses had developed a technique to be quicker, resulting in a shorter feed intake time during the last 2 days of the experiment. The question that remains to be answered in our study is if the horses would adapt to quicker eating from the hayball and the haynet, thus evening out the measured differences in feed intake time. Glunk et al. [26] studied a haynet with three different hole sizes and found that the feed intake time was shorter for the largest hole size (15.2 cm openings) when compared to medium (4.2 cm) and smaller (3 cm) openings, thus showing that the hole size of haynets affects the feed intake time. In our study, the hole size of the haynet was 10 cm in diameter and 7 cm for the hayball, which is in between the hole sizes in the study of Glunk et al. [26], and in our study, the feed intake time from the haynet and the hayball was 49 and 51 min/kg haylage, respectively, compared to 43 min/kg in the largest openings and 56 min for the medium openings in the Glunk et al. study [26]. Therefore, it can be concluded that the diameter of the holes/openings of the slow feeding equipment seems to influence the intake rate similarly (in our and Glunk et al. studies), despite different forages and sizes of horses. Further studies are needed regarding the feed intake time of Icelandic horses using different types of forages, e.g., DM%, botanical composition, and maturity stage. Additionally, it is important to study the effects of different body postures and movements when using different feeding methods, e.g., haynets, as Raspa et al. [37] found that different heights of the haynet from the floor can affect the position of the horse’s neck and back during eating. The height of the haynet in our study was relatively similar to the high-positioned haynet in the study of Raspa et al., [37] (the bottom edge being at elbow height of the horses studied) and the top of the manger was even higher. These high positions put the horse in an unnatural eating position, making them keep their neck above the withers and keep the mandibular angle smaller than in a natural eating position. It should be kept in mind when finding proper feeding methods to extend the feed intake time of stabled horses, the natural body posture of horses when eating is to graze with the head and neck in a downward position. In our study, the hayball was closer to the floor than the haynet, allowing a more “natural eating posture”, and the feed intake time was similar to the haynet, although more research is surely needed. Because the height of the haynet and hayball are relative to the horses’ size, it is important to be aware of that our results concern Icelandic horses (height at withers ~142 cm), and the use of these devices could provide different results in both larger and/or smaller equids. Additionally, the forces needed to pull the haylage out were not evaluated in our study. It has been stressed [38] that the forces and body movements needed by horses to extract hay from the haynet holes could possibly cause wear and tear to teeth, muscles, joints, and bones, and, therefore, it has been proposed [38] that more research is needed regarding the use of haynets for feeding horses. For practical reasons, it is possible that filling up many hayballs in larger stables is unfeasible, as it is quite labour intensive (2½ to 3 min per hayball in this study). The limitations of our study include the fact that it is small and limited by the number of horses, experimental days, and only one forage type being used. It would also have been of interest to measure how long the experimental horses spend grazing when allowed free access as a reference point for comparison.

## 5. Conclusions

From this study, it can be concluded that feeding high-energy haylage in a hayball or in a haynet can increase the feed intake time of Icelandic horses by, on average, 13% per day when compared to feeding it in a metal corner manger or on the box floor. Thus, it is possible, with simple feeding methods, to extend the feed intake time of maintenance-fed horses in order to closer resemble their natural foraging time.

## Figures and Tables

**Table 1:** Dry matter (DM) content (g/kg), DM digestibility (%), and chemical composition (g/kg DM) of the experimental haylage evaluated using NIRS [32].

Dry Matter	542
DM digestibility % ^1^	72
Fu/kg DM ^2^	0.75
Crude protein	144
Digestible crude protein ^3^	95
NDF	524
Sugars	70

^1^ Calibrated for ruminants. ^2^ Fu = Feed unit (net energy, 1650 kcal). ^3^ Calculated according to ((CP/10 × 0.891–3.31) × 10).

**Table 2:** Average feed intake time (LSmeans ± SE) of haylage in minutes (min) per kg of dry matter and per meal (3.5 kg, 54.2% DM) in 4 adult Icelandic horses, using 4 different feeding methods: metal corner manger, box floor, haynet, and hayball.

Average Feed Intake Time	Metal Manger	Box Floor	Haynet	Hayball	SE
Per kg DM, min	81 ^a^	85 ^a^	94 ^b^	96 ^b^	±2
Per meal, min	154 ^a^	160 ^a^	178 ^b^	183 ^b^	±4.5

Numbers with different superscripts within same row differ *p* < 0.05

## Data Availability

Data available on request from the authors.

## References

[1] Janis C. (1976). The evolutionary strategy of the Equdae and the origins of rumen and caecal digestion. Evolution.

[2] Ellis A.D., Ellis A.D., Longland A.C., Coenen M., Miraglia N. (2010). Biological basis of behaviour in relation to nutrition and feed intake in horses. The Impact of Nutrition on the Health and Welfare of Horses.

[3] Boyd L.E., Carbonaro D.A., Houpt K.A. (1988). A 24-hour time budget of Przewalski horses. Appl. Anim. Behav. Sci..

[4] McGreevy P. (2004). Equine Behavior. A Guide for Veterinarians and Equine Scientists.

[5] Hothersall B., Nicol C.J., Geor R.J., Harris P.A., Coenen M. (2013). Effects of diet on behaviour, -normal and abnormal. Equine Applied and Clinical Nutrition.

[6] Wickens C.L., Heleski C.R. (2010). Crib-biting behavior in horses: A review. Appl. Anim. Behav. Sci..

[7] Henderson A.J.Z. (2007). Don’t fence me in: Managing psychological well being for elite performance horses. J. Appl. Anim. Welf. Sci..

[8] Normando S., Meers L., Samuels W.E., Faustini M., Ödberg F.O. (2011). Variables affecting the prevalence of behavioural problems in horses. Can riding style and other management factors be significant?. Appl. Anim. Behav. Sci..

[9] Harris P.A., Ellis A.D., Fradinho M.J., Jansson A., Julliand V., Luthersson N., Santos A.S., Vervuert I. (2017). Feeding conserved forage to horses: Recent advances and recommendations. Animal.

[10] Sarrafchi A., Blokhuis H.J. (2013). Equine stereotypic behaviors: Causation, occurrence, and prevention. J. Vet. Behav.-Clin. Appl. Res..

[11] Sneddon J.C., Argenzio R.A. (1998). Feeding strategy and water homeostasis in equids: The role of the hind gut. J. Arid. Environ..

[12] Halldórsson P. (2001). Hestafóður. Stöðumat á Fóðrun Reiðhesta. [e. Feeds for Horses. Status of Riding Horses’ Feeding]. Bachelor’s Thesis.

[13] Müller C.E. (2011). Equine ingestion of haylage harvested at different plant maturity stages. Appl. Anim. Behav. Sci..

[14] Ragnarsson S., Lindberg J.E. (2008). Nutritional value of timothy haylage in Icelandic horses. Livest. Sci..

[15] Lindberg J.E., Geor R.J., Harris P.A., Coenen M. (2013). Feedstuffs for horses. Equine Applied and Clinical Nutrition.

[16] Ragnarsson S., Lindberg J.E. (2010). Nutritional value of mixed grass haylage in Icelandic horses. Livest. Sci..

[17] NRC (2007). Nutrient Requirements of Horses.

[18] Ragnarsson S. (2009). Digestibility and Metabolism in Icelandic Horses Fed Forage-Only Diet. Ph.D. Thesis.

[19] Ragnarsson S., Jansson A. (2011). Comparison of grass haylage digestibility and metabolic plasma profile in Icelandic and Standardbred horses. J. Anim. Physiol. Anim. Nutr..

[20] Stefánsdóttir G. (2010). Útifóðrun hrossa. [e. Feeding horses outside]. Eiðfaxi.

[21] Wyse C.A., McNie K.A., Tannahil J.K., Murray J.K., Love S. (2008). Prevalence of obesity in riding horses in Scotland. Vet. Rec..

[22] Jensen R.B., Danielsen S.H., Tauson A.H. (2015). The prevalence of obesity in mature Icelandic horses in Danmark. Acta Vet. Scand..

[23] Pratt-Phillips S., Munjizun A. (2023). Impacts of Adiposity on Exercise Performance in Horses. Animals.

[24] Lundqvist H., Müller C.E. (2022). Feeding time in horses provided roughage in different combinations of haynets and on the stable floor. Appl. Anim. Behav. Sci..

[25] Ellis A.D., Redgate S., Zinchenko S., Owen H., Barfoot C., Harris P. (2015). The effect of presenting forage in multi-layered haynets and at multiple sites on night time budgets of stabled horses. Appl. Anim. Behav. Sci..

[26] Glunk E.C., Hathaway M.R., Weber W.J., Sheaffer C.C., Martinson K.L. (2014). The effect of hay net design on rate of forage consumption when feeding adult horses. J. Equine Vet. Sci..

[27] Rochais C., Henry S., Hausberger M. (2018). “Hay-bags” and “Slow feeders”: Testing their impact on horse behaviour and welfare. Appl. Anim. Behav. Sci..

[28] Correa M.G., e Silva C.F.R., Dias L.A., Junior S.D.S.R., Thomes F.R., do Lago L.A., Carvalho A.D.M., Faleiros R.R. (2020). Welfare benefits after the implementation of slow-feeder hays bags for stabled horses. J. Vet. Behav.-Clin. Appl. Res..

[29] Ellis A.D., Fell M., Luck K., Gill L., Owen H., Briars H., Barfoot C., Harris P. (2015). Effect of forage presentation on feed intake behaviour in stabled horses. Appl. Anim. Behav. Sci..

[30] Stefánsdóttir G.J., Björnsdóttir S. (2001). Mat á holdafari hrossa [e. Estimation of horses’ body condition]. Eiðfaxi-Ræktun.

[31] Henneke D.R., Potter G.D., Kreider J.L., Yeates B.F. (1983). Relationship between condition score, physical measurements and body fat percentage in mares. Equine Vet. J..

[32] (2012). Efnagreining.is. https://efnagreining.is/.

[33] Austbø D., Udén P., Eriksson T., Müller C.E., Spörndly R., Liljeholm M. (2010). Energy and protein requirements for horses in the Nordic countries. Proceedings of the 1st Nordic Feed Science Conference.

[34] Benz B., Münzing C., Krüger K., Winter D. (2014). Ethological investigation of hayracks in equine husbandry. Landtechnik.

[35] Waters A.J., Nicol C.J., French N.P. (2002). Factors influencing the development of stereotypic and redirected behaviours in young horses: Findings of a four year prospective epidemiological study. Equine Vet. J..

[36] Whisher L., Raum M., Pina L., Pérez L., Erb H., Houpt C., Houpt K. (2011). Effects of environmental factors on cribbing activity by horses. Appl. Anim. Behav. Sci..

[37] Raspa F., Roggero A., Palestrini C., Marten Canvesio M., Bergero D., Valle E. (2021). Studying the Shape Variations of the back, the neck, and the Mandibular Angle of Horses Depending on Specific Feeding Postures Using Geometric Morphometrice. Animals.

[38] Hodgson S., Bennett-Skinner P., Lancaster B., Upton S., Harris P., Ellis A.D. (2022). Posture and pull pressure by horses when eating hay or haylage from a hay net hung at various positions. Animals.

